# Infestation ratings database for soybean aphid on early-maturity wild soybean lines

**DOI:** 10.1016/j.dib.2017.09.012

**Published:** 2017-09-12

**Authors:** Louis S. Hesler, Kelley J. Tilmon

**Affiliations:** a USDA-ARS, North Central Agricultural Research Laboratory, Brookings, SD 57006, USA; b Department of Entomology, The Ohio State University, Ohio Agricultural Research and Development Center, 1680 Madison Ave., Wooster, OH 44691, USA

**Keywords:** Crop ancestors, Resistance screening, Host-plant resistance

## Abstract

Soybean aphid (*Aphis glycines* Matsumura; SA) is a major invasive pest of soybean [*Glycine max* (L.) Merr.] in northern production regions of North America. Although insecticides are currently the main method for controlling this pest, SA-resistant cultivars are being developed to sustainably manage SA in the future. The viability of SA-resistant cultivars may depend on identifying a diverse set of resistance genes from screening various germplasm sources, including wild soybean (*Glycine soja* Siebold and Zucc.), the progenitor of cultivated soybean. Data consisted of infestation ratings generated for a total of 337 distinct plant introduction lines of wild soybean that were exposed to avirulent SA biotype 1 for 14 d in 25 separate tests. Individual plants of the test lines were given a common rating by two researchers, based on a rating scale that progressed from 1=0 to 50, 2=51 to 100, 3=101 to 150, 4=151 to 200, 5=201 to 250, and 6 with >250 SA per test plant. Public dissemination of this dataset will allow for further analyses and evaluation of resistance among the test lines.

**Specifications Table**

**Table:** 

Subject area	*Biology*
More specific subject area	*Host-plant resistance*
Type of data	*Table, figure*
How data was acquired	*Rating infestations on a 1-to-6 scale*
Data format	*Raw*
Experimental factors	*Plant introduction lines of wild soybean,* Glycine soja*; soybean aphid biotype 1*
Experimental features	*Two-week-old plants of various wild soybean lines were subjected to infestation by soybean aphids in a no-cage choice test, and infestations were rated 14 d later.*
Data source location	*USDA-ARS, Brookings, SD USA*
Data accessibility	*Data are available with this article*
Related research article	*Not applicable.*

**Value of the data**

• Useful in identifying plant introduction (PI) lines of wild soybean with putative resistance to soybean aphid (SA).
• Provides baseline dataset with avirulent SA biotype 1 that can be compared in future tests against virulent SA biotypes.
• Useful in deciding what PI lines to include in follow-up resistance tests with SA.
• Provides phenotypic data that can be used in studies that genetically characterize the basis of resistance in soybean.

## 1 Data

Infestation ratings for individual plants of various wild soybean lines following 14 d of exposure to SA.

## 2 Experimental design, materials and methods

### 2.1 Overview

Altogether, 337 distinct plant introduction (PI) lines of wild soybean were evaluated against avirulent SA biotype 1 in environmental chambers (Conviron, CMP4030, Winnipeg, Canada) at the USDA-ARS North Central Agricultural Research Laboratory, Brookings, SD. Tests were run at 16:8 L:D photoregime and temperatures from 18 °C (nighttime) to 22 °C (daytime). Lines were evaluated time in 25 no-cage choice tests using procedures adapted from Hesler [1] and Hesler et al. [2]; some test lines, especially those that showed resistance in one test, were repeated in a second test.

### 2.2 Test lines

Test lines were early maturity (maturity group I) germplasm obtained from the USDA-ARS Soybean Germplasm Collection, National Soybean Research Center, Urbana, IL, USA. Individual test lines were identified with their respective PI numbers or with a number beginning with the digits ‘99’ to indicate a 1999 seed lot in which the original PI number could not be verified.

### 2.3 Test procedures

The test plants were prepared by placing two seeds of a test line into a cylindrical peat pellet (36 mm diam; Ferry-Morse Seed Co., Fulton, KY, USA), and the seeded pellet was then saturated with water. Ten to 12 d later, pellets were thinned to one seedling and transferred to individual 8.5-cm square plastic pots and filled with a 2:1:1 mixture of soil, vermiculite, and peat moss. The soil surface of each pot was covered with 2-cm of sand to provide a more even surface for aphid dispersal and to preempt fungus gnat infestation in the soil.

**Fig. 1:**
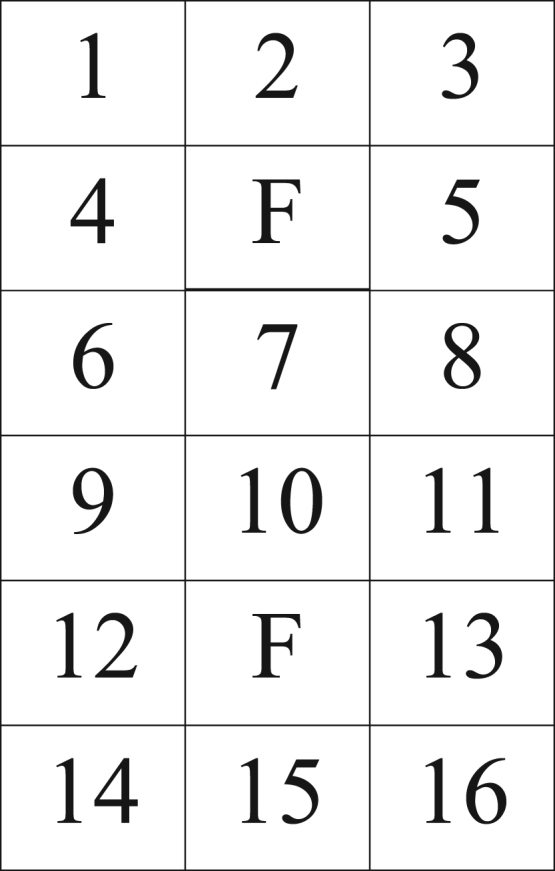
Layout for screening 16 *Glycine soja* lines (14 test lines and two checks) in a replicate tray with two spaces for aphid-infested founder plants (F) equidistant from immediately surrounding test lines, which were randomly assigned to each numbered space.

Test lines were arranged with a RCBD in 24 of the tests. Fourteen potted test lines were placed into a plastic tray along with a susceptible check (99PI423993 in the first 8 tests and test #13; PI 522212 B in the last 17 tests), a resistant check (PI 549046), and two aphid-source plants. No resistant check was available in test #s 5, 6, and 7, and thus a different test line was added in each of those tests. A source plant was placed at one of two foci in the tray that were placed equidistant from immediately surrounding test lines to facilitate even dispersion of aphids (Fig. 1). Each source plant was a 4-wk-old soybean plant that was infested with about 250 SA biotype 1; the type of source plant corresponded to the rearing plant used at the time, i.e. ‘SD1091(RR)’ for the first four tests and PI 667735 (‘Brookings’) thereafter. SA were reared as a colony established from field collections in 2009 near Brookings, South Dakota, USA. Each tray comprised a replicate, and 6–8 replications were used per test. Due to limited numbers of the resistant check from poor germination, test #13 consolidated 33 lines, both susceptible checks, and the resistant check among 18 trays using a CRD; each tray had source plants at two foci equidistant from test lines and checks.

Source plants were clipped at soil level at the start of each test to induce wilting and subsequent dispersal of SA to test plants and checks, and the clipped plants were supported by attaching them via paper clip to a 10-cm long, 3-mm diameter wooden stake inserted into the soil. After 14 d, test lines in were given a common rating by two researchers, based on a rating scale: ‘1’=0 to 50, ’2’=51 to 100, ‘3’=101 to 150, ‘4’=151 to 200, ‘5’=201 to 250, and ‘6’ with >250 SA per test plant.

## Funding sources

The study was funded by USDA-ARS Project 5447-21220-005-00D, the South Dakota Soybean Research and Promotion Council Project, SA1500518 and the North Central Soybean Regional Project SA1400620.

## References

[1] Hesler L.S. (2013). Resistance to soybean aphid among wild soybean lines under controlled conditions. Crop Prot..

[2] Hesler L., Beckendorf E., Schultz N., Van De Stroet B., Tilmon K., Rozeboom P. (2017). Resistance to soybean aphid in early maturing plant introduction lines of soybean, 2012–2015. Arthro. Manag. Tests.

