# Supplementary material for: Infestation ratings database for soybean aphid on early-maturity wild soybean lines
Source: Data Brief. 2017 Sep 12;15:138–41. doi: 10.1016/j.dib.2017.09.012 (PMC5972814; doi:10.1016/j.dib.2017.09.012)
Supplement: Supplementary file 1 — Transparency document [file mmc1.docx]

**Manuscript Title:** Infestation ratings database for soybean aphid on early-maturity wild soybean lines

**Authors:** Louis S. Hesler, Kelley J. Tilmon

The authors certify that they have NO affiliations with or involvement in any organization or entity with any financial interest (such as honoraria; educational grants; participation in speakers’ bureaus; membership, employment, consultancies, stock ownership, or other equity interest; and expert testimony or patent-licensing arrangements), or non-financial interest (such as personal or professional relationships, affiliations, knowledge or beliefs) in the subject matter or materials discussed in this manuscript.
